# P-2239. RNA Signatures for Diagnosing or Predicting Febrile Illness: A Descriptive Analysis

**DOI:** 10.1093/ofid/ofae631.2392

**Published:** 2025-01-29

**Authors:** Hannah N Kozlowski, Rebecca Womersley, Sam Brophy-Williams, Costanza Di Chiara, Ryan S Huang, Nikki Wong, Katie Lee, Joelle Peresin, Shaun Morris

**Affiliations:** University of Toronto, Toronto, Ontario, Canada; Imperial College London, London, England, United Kingdom; Hospital for Sick Children, Toronto, Ontario, Canada; The Hospital for Sick Children, Toronto, Ontario, Canada; University of Toronto Temerty Faculty of Medicine, Toronto, Ontario, Canada; Temerty Faculty of Medicine, University of Toronto, Markham, Ontario, Canada; University of Toronto Temerty Faculty of Medicine, Toronto, Ontario, Canada; Hospital for Sick Children, Toronto, Ontario, Canada; Hospital for Sick Children, University of Toronto, Toronto, Ontario, Canada

## Abstract

**Background:**

Fever is a common sign of illness; it may self-resolve or be present in serious illnesses requiring hospitalization. Investigating the cause of fever can be extremely resource intensive requiring multiple diagnostic tests and healthcare providers thereby delaying time to diagnosis. RNA molecules present in the blood can be easily detected and reflect a patient’s disease state, making them ideal biomarkers.

Our objective was to identify RNA common to signatures that diagnose and predict the outcome of febrile illnesses. Our secondary objectives were to assess whether signatures are being validated and translated.

**Methods:**

We conducted a scoping review of published research articles, including preprints, from EMBASE and Medline that measured blood RNA in patients with febrile illness. Here we are analyzing a articles that used array or RNA-seq to generate original signatures.

**Results:**

We identified 248 original blood RNA signatures for the diagnosis or prognosis of febrile illness. 154 were diagnostic, 56 prognostic, 15 diagnostic/prognostic and 23 exploratory. The most common illnesses studied were sepsis (n=83), viral infections (n=74) and tuberculosis (n=38). From all sepsis signatures there were 214 unique RNA and 74 RNA present in multiple signatures. The most common RNA, C3AR1 (complement receptor) and MPO (myeloperoxidase), appeared in 5 signatures. For viral infections there were 54 signatures, and IFI27 (interferon) appeared in 8 signatures. For tuberculosis there were 26 signatures, and the most common RNA were BATF2 (transcription factor) and FCGR1B (Fc receptor) each appearing in 4 signatures.

158/248 signatures were validated. Clinical sensitivity and specificity in validation groups were 54-100% and 79-97%, respectively. 9/248 studies claimed that they had filed or were filing for a patent.

**Conclusion:**

Blood RNA molecules can be used for the diagnosis and prognosis of febrile illnesses. Currently, < 65% of RNA signatures are validated and < 5% of signatures are commercialized. In conducting this analysis, we encountered a lack of standardization in methods reporting. Further work to improve transparency and encourage validation is needed so RNA signatures can be adapted into simple, molecular diagnostic tests and translated for patient use.

**Disclosures:**

Shaun Morris, MD, MPH, DTM&H, FRCPC, FAAP, GSK Canada: Advisor/Consultant|GSK Canada: Honoraria|Pfizer: Advisor/Consultant|Pfizer: Honoraria|Sanofi Canada: Advisor/Consultant|Sanofi Canada: Honoraria

